# Scoring colorectal cancer risk with an artificial neural network based on self-reportable personal health data

**DOI:** 10.1371/journal.pone.0221421

**Published:** 2019-08-22

**Authors:** Bradley J. Nartowt, Gregory R. Hart, David A. Roffman, Xavier Llor, Issa Ali, Wazir Muhammad, Ying Liang, Jun Deng

**Affiliations:** 1 Department of Therapeutic Radiology, School of Medicine, Yale University, New Haven, Connecticut, United States of America; 2 Sun Nuclear Corporation, Melbourne, FL, United States of America; 3 Department of Digestive Diseases, School of Medicine, Yale University, New Haven, Connecticut, United States of America; University of Munich, GERMANY

## Abstract

Colorectal cancer (CRC) is third in prevalence and mortality among all cancers in the US. Currently, the United States Preventative Services Task Force (USPSTF) recommends anyone ages 50–75 and/or with a family history to be screened for CRC. To improve screening specificity and sensitivity, we have built an artificial neural network (ANN) trained on 12 to 14 categories of personal health data from the National Health Interview Survey (NHIS). Years 1997–2016 of the NHIS contain 583,770 respondents who had never received a diagnosis of any cancer and 1409 who had received a diagnosis of CRC within 4 years of taking the survey. The trained ANN has sensitivity of 0.57 ± 0.03, specificity of 0.89 ± 0.02, positive predictive value of 0.0075 ± 0.0003, negative predictive value of 0.999 ± 0.001, and concordance of 0.80 ± 0.05 per the guidelines of Transparent Reporting of Multivariable Prediction Model for Individual Prognosis or Diagnosis (TRIPOD) level 2a, comparable to current risk-scoring methods. To demonstrate clinical applicability, both USPSTF guidelines and the trained ANN are used to stratify respondents to the 2017 NHIS into low-, medium- and high-risk categories (TRIPOD levels 4 and 2b, respectively). The number of CRC respondents misclassified as low risk is decreased from 35% by screening guidelines to 5% by ANN (in 60 cases). The number of non-CRC respondents misclassified as high risk is decreased from 53% by screening guidelines to 6% by ANN (in 25,457 cases). Our results demonstrate a robustly-tested method of stratifying CRC risk that is non-invasive, cost-effective, and easy to implement publicly.

## Introduction

Colorectal adenocarcinomas are the result of unregulated growth in the colon mucosa that commonly starts with polypoid lesions progressing into advanced cancers [1]. Of all new cancer cases in the US, 8.0% are colorectal. Colorectal cancer (CRC) claims 8.4% of all cancer-deaths and the overall 5-year survival rate is 66% [2]. Early stage (localized) CRC has a 5-year survival rate close to 90%, while that of distant/metastatic CRC survival is less than 14% [2]. Treatment of colorectal cancer even in its metastatic state is fairly standardized, typically with bevacizumab [3] which has a side-effect of high blood pressure dependent upon the dose [4,5].

There are multiple personal health factors correlating moderately with incidence of advanced colorectal neoplasia [6] and colorectal cancer which are both self-reportable, easily gathered, and usable in scoring CRC risk [7–10]. For example, CRC is more frequent in men than women and African Americans have the highest incidence in the US [2,11]. Environmental factors, socio-economic features, and co-morbidities additionally influence CRC risk [2]. In a study of subjects from Wisconsin and Minnesota, risk factors for long-term colorectal-cancer mortality were found to be age, sex, and higher body-mass index (BMI) [12], though colorectal-cancer incidence was only marginally increased with higher BMI. In a meta-study more specialized to BMI as a factor of CRC risk, those of lower BMI were at risk for colorectal cancer as well [13]. It is possible to build a CRC risk score from these many factors, and many authors have done so [14–16].

**Fig 1:**
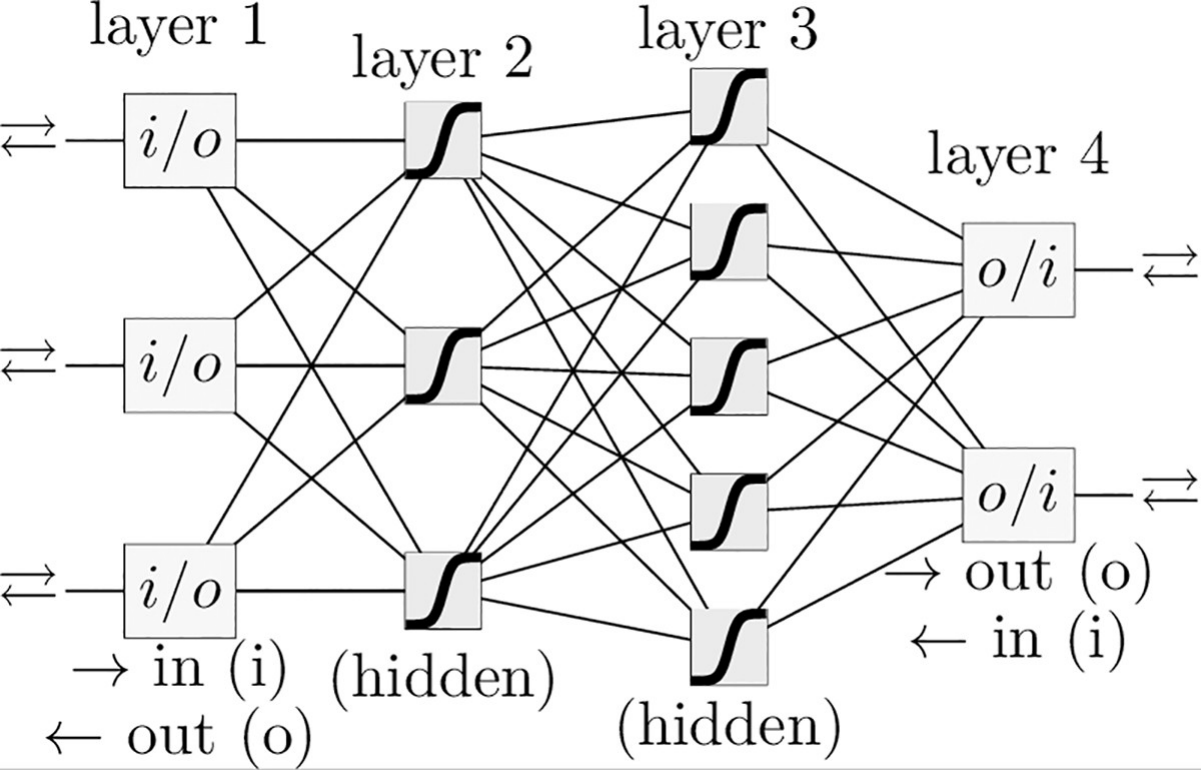
Schematic of example ANN. A schematic of an ANN with four layers and a logistic activation function. The ANN in this paper has one input neuron for each of the 12 to 14 factors, the same number of neurons in each hidden layer, and a single output neuron for the prediction. The upper arrow indicates forward-propagation and the lower arrow indicates back-propagation.

Efforts to create risk indexes or prediction models for CRC [14–16] have used a variety of data of three types: (1) routine, (2) reportable by self-completed questionnaire, (3) genetic biomarkers as recently summarized [7,17]. While logistic regression [18] is a popular method of scoring CRC risk [15], this work opts to use an artificial neural network (ANN) trained with professionally-collected routine data [19] and shown in Fig 1. While an ANN is not strictly superior to logistic regression [20], an ANN incorporates complicated inter-factor coupling [21]. Given the complexity of human biology, inter-factor coupling is likely to be important in the predicting CRC from personal health data.

The United States Preventative Services Task Force [22] (USPSTF) and various medical societies [23] currently recommend screening by age and family history only, despite models incorporating additional risk factors. Specifically, those with no family history of cancer aged 50–75 are recommended for screening [22,24], meaning that United States citizens living to age 50 and beyond are flagged for screening. Of the incidences of CRC in the National Health Interview Survey (NHIS) dataset [19] within 4 years of the survey, 65.6% occur in ages 50–75 and 80.7% are ages 50 and older, leaving 19.3% under age 50 and outside the USPSTF screening guidelines. The USPSTF’s screening guidelines saves many lives [25] but at the expense of many false-positives, which could lead to unnecessary, expensive, and occasionally injurious screening. There is also a remainder of false-negatives (specifically, CRC occurring under age 50 in those with no family history [11]) that could be flagged for screening by an appropriate model of risk.

Current screening procedures for CRC (by colonoscopy [24,26,27] every 10 years or sigmoidoscopy [28,29] / colonography [22] every 5 years) are often invasive with suboptimal accuracy, and always expensive. For example, perforation of the colon and/or bleeding during both colonoscopy and flexible sigmoidoscopy have been reported, demonstrating the need for non-invasive techniques [22]. Hence, even at the expense of lowered positive/negative predictive value (PPV/NPV), there has been a push for less expensive and less invasive screening methods. From the 1990s to the 2010s, this push has yielded the yearly fecal occult blood test (FOBT) [29] and the yearly fecal immunochemical test (FIT) [30] possibly combined [31] with the SEPT9-methylation [32,33] test. An efficient and non-invasive method that can help clinicians identify appropriate CRC screenings for individuals (e.g., colonoscopy/sigmoidoscopy for high risk and stool tests for low risk) is desired [34,35].

In this work, we use an artificial neural network (ANN) to score an individual’s risk of CRC as a means of assisting screening recommendations. Previously, the ANN has been used to assist diagnosis from expertly-collected data (e.g., distinguishing between sporadic colon adenomas and cancers vs. inflammatory bowel disease-related dysplasia or cancer) from high-dimensional genetic datasets [14]. This application is limited to use by professionals. In contrast, a comparatively-large portion of the population can self-report their habits of smoking, exercise, their hypertension, diabetes, emphysema, and other personal-health data. Our ANN is trained with this latter data specifically because of the ease of self-reporting per TRIPOD guidelines [36]. This makes it inappropriate for use as a diagnostic tool, but able to be mass-implemented. Members of the general public can make personal decisions to be screened [22] based on their score of CRC risk, and clinicians can use this same score to assist their screening recommendations.

## Materials and methods

### Data

The data to train and validate the ANN was the 1997–2016 responses to the NHIS sample adult questionnaire from the Centers for Disease Control and Prevention (CDC) [19]. About 20% of respondents were discarded in our study due to missing entries in the NHIS questionnaire. The NHIS data inquired about both colon and rectal cancer. Therefore, we are counting anyone that had colon cancer, rectal cancer, or both colon and rectal cancer as a single incidence of colorectal cancer. Subjects who were diagnosed with CRC more than 4 years prior to the time of survey were discarded from the dataset and thus not used. While the NHIS dataset records the age at which the respondent was professionally diagnosed with CRC (if at all), the dataset does not record the time at which diagnoses of other predictors (e.g., diabetes) was given. Therefore, to increase the probability that the predictors arose prior to the cancer we only include those that were recently diagnosed with cancer (within four years prior to taking the NHIS).

**Fig 2:**
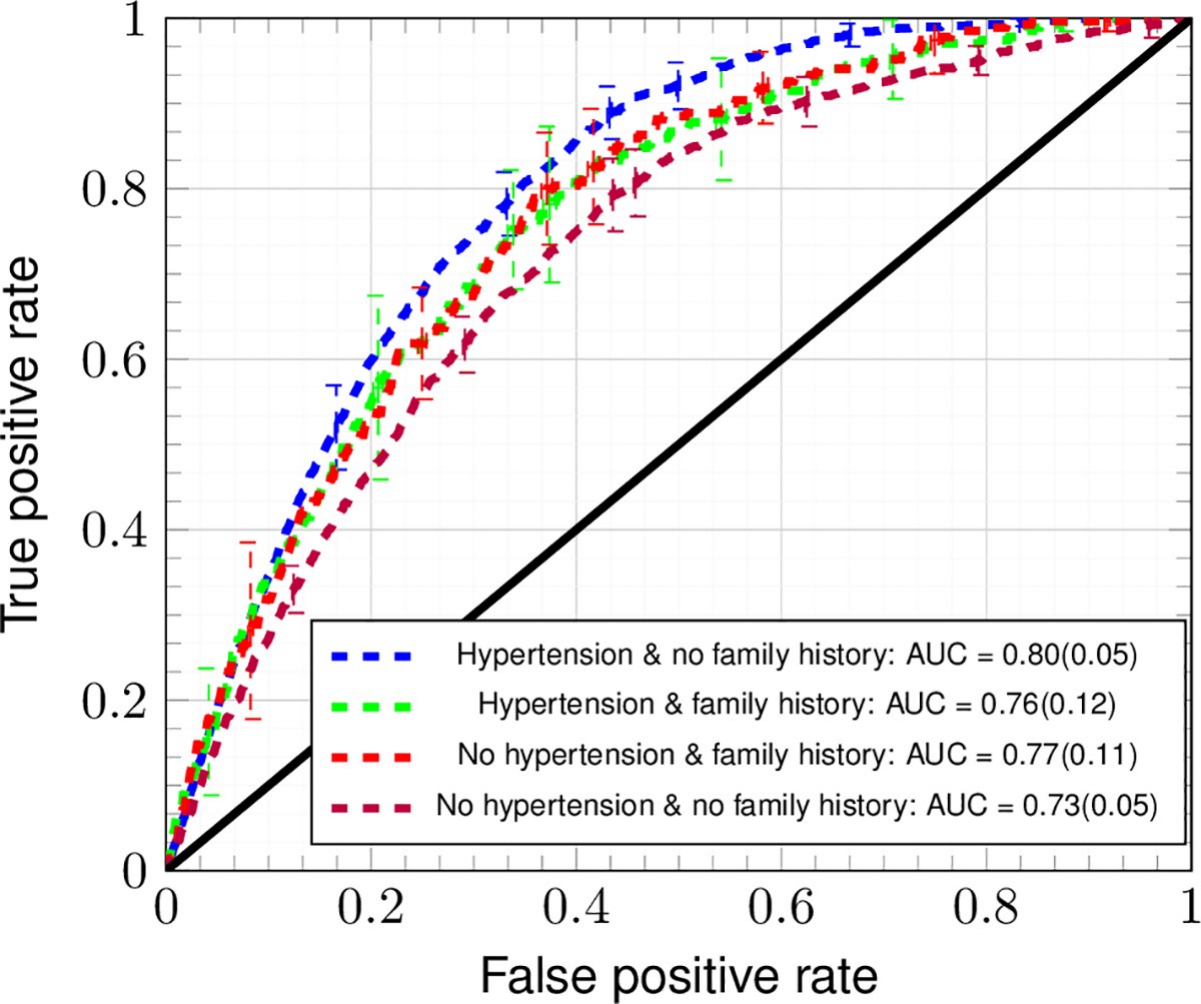
ROC curves of the ANN for ten-fold cross-testing dataset (TRIPOD 2a). The ANN trained with the factors marked “default model” in Table 2 with (blue line) and without hypertension (purple line). The ANN was also trained on a reduced dataset that included family history with (green line) and without (red line) hypertension. Error bars denote the standard deviation of the TPR and FPR across ten folds of stratified cross-testing (TRIPOD level 2a).

For the default model, 525,394 and 58,376 subjects were used to train and test the ANN respectively, among whom 1,269 and 140 respondents were told by a doctor or other health professional that they had CRC recently; that is, within 4 years of their taking the NHIS. (This is referred to as “recently and professionally diagnosed with CRC” throughout the manuscript)recently and professionally diagnosed with CRC. Variants of this model whose resulting ROCs are studied in Fig 2 significantly change the number of subjects in each population. Each population is given in Table 1.

**Table 1:** Number of NHIS respondents in final dataset when certain factors are chosen.

Ever screened? †	Family history†	Age (years)	Hyper- -tension	Training	Training & CRC	Testing	Testing& CRC
Unused◊	Unused◊	18–85◊	Used◊	525,394◊	1,269◊	58,376◊	140◊
Unused	Used	18–85	Used	105,950	245	11,772	27
Unused	Used	18–85	Unused	105,760	245	11,723	27
Unused	Unused	18–85	Unused	525,394	1,269	58,376	140
Unused	Unused	18–49	Used	298,085	162	33,120	18
Unused	Unused	50–75	Used	227,310	1,107	25,255	122
Used	Unused	18–85	Used	10,261	72	217,774	446
Used	Used	18–85	Used	66,938	300	161,098	218
Used	Used	18–85	Unused	9,002	59	219,176	459
Used	Unused	18–85	Unused	13,755	71	212,995	443

† Data appearing in NHIS years 2000, 2005, 2010, and 2015 only when a set of supplementary questions were asked.

◊ Data in the default model.

From the NHIS data we obtained the factors appearing in Table 2. Heart conditions are pooled into 1 factor, and training is done with and without data on hypertension and CRC family history, leaving 12 to 14 factors. These factors were selected because they correlate strongly with CRC incidence and also have either “ever” as their time of incidence (see Discussion), are “permanent” (e.g., age, ethnicity), or are “per week” (vigorous exercise) frequency. The relative importance of these factors (in terms of Pearson correlation [37] with CRC) are presented in Table 2.

**Table 2:** All factors in the NHIS datasets used to train the ANN in scoring CRC risk, in descending order of correlation magnitude.

Name of Factor	Correlation with Recent CRC, ×10^−2^	Type of Factor	# of Unique Values of Factor	Time of Incidence, Frequency, or Duration
Current or Cancer Age†	+4.907	Continuous	68	Permanent
Hypertension†	+3.045	Ordinal	2	Ever
Number of first-degree relatives with CRC (NHIS years 2000, 2005, 2010, and 2015 only)	+2.906	Ordinal	4	Permanent
Coronary heart disease	+2.349	Ordinal	2	Ever
Pooled heart conditions†	+2.063	Ordinal	2	Ever
Myocardial infarction	+2.060	Ordinal	2	Ever
Diabetes (non-gestational) †	+2.056	Ordinal	3	Ever
Heart condition/disease	+1.972	Ordinal	2	Ever
Vigorous exercise frequency†	-1.971	Continuous	33	Per week
Angina pectoris	+1.769	Ordinal	2	Ever
Ulcer (stomach, duodenal, peptic)†	+1.540	Ordinal	2	Ever
Hispanic ethnicity†	-1.269	Categorical	2	Permanent
Stroke†	+1.218	Ordinal	2	Ever
Emphysema†	+1.220	Ordinal	2	Ever
American Indian, African American, other, or multiple race †	-0.494	Categorical	2	Permanent
Sex (male) †	-0.350	Categorical	2	Permanent
Body-mass index†	+0.234	Continuous	4223	Current
Smoking frequency†	+0.0461	Ordinal	4	Current

† Denotes factors that are part of the model referred to as “default” throughout this paper.

One of the 14 factors we would like to use is hypertension, but the approval [38] of bevacizumab as a treatment for CRC in 2004 can result in many people having hypertension because of the treatment of CRC instead of being a risk factor for CRC itself. Therefore, we determined the correlation of hypertension and CRC prior to 2004 (3.16×10^−2^) and after 2004 (2.86×10^−2^). This is an unexpected post-approval [3,38] decrease of 3×10^−3^. Thus, we decided bevacizumab-induced hypertension was unimportant and we used hypertension in all models unless indicated otherwise.

Raw data in the NHIS dataset was mapped to the interval [0,1] to be input to the ANN in two ways depending on whether the data is categorical or ordinal. Referring to Table 2, the factors of having hypertension, ulcers, a stroke, and emphysema are binary variables and naturally to map to 0 for “no”, 1 for “yes”. Diabetic status can have one of three discrete values: not diabetic, pre-diabetic/borderline, and diabetic. These were mapped to 0, 0.5, and 1, respectively. The age factor is continuous and is the age at the time of responding to the NHIS if the respondent never had any cancer or the age at which they were recently and professionally diagnosed with CRC otherwise. The factors of weekly frequency of vigorous exercise and BMI are also continuous. The NHIS defines vigorous exercise as lasting 10 minutes or more and resulting in one or more of: heavy sweating, breathing, or elevated heart rate. All such continuous factors are unitized to the interval [0,1] using the replacement $x\Rightarrow \frac{x-\min x}{\max x-\min x}$ for factor *x*. The factor sex is 0 for women and 1 for men. The variable of Hispanic ethnicity was given a value of 0 for a response of “Not Hispanic/Spanish origin” and 1 otherwise. The variable of race was set to 1 for responses of “Black/African American only”, “American Indian only”, “Other race”, or “Multiple race” and 0 otherwise (avoiding one-hot encoding to reduce overfitting). The smoking status had a value of 1 for an everyday smoker, 0.66 for a some-day smoker, 0.33 for a former smoker, and 0 for a “never smoker”. The NHIS defines a “never smoker” as one who has smoked 100 cigarettes or less over their entire life, and a “former smoker” as a smoker who has quit at least 6 months. The variable of family history is the number of first-degree relatives with CRC but is capped at 3, which is then mapped to values of 0, 1/3, 2/3, 1. Finally, any answer of “yes” to coronary heart disease, myocardial infarction, heart disease, and angina contributes 0.25 to the “Pooled heart conditions” field. These mapped values (rather than the raw data) are used in Table 2 for the correlation calculation.

### An artificial neural network (ANN)

An artificial neural network is a network of neurons, with each neuron being equivalent to a logistic regression. A neuron’s inputs, the outputs of the preceding layer’s neurons, are combined in a weighted sum with a bias term. This linear function is fed into a sigmoidal (“activation”) function to produce the neuron’s output. The input layer consists of the model’s input data (the NHIS data). The output layer returns model predictions to the user. In this work, our ANN has only one neuron in the output layer, representing an individual’s risk score for CRC. Layers between the input and output layer are known as hidden layers. Therefore, an ANN is essentially a statistical regression that is nonlinear with respect to the model parameters. Note that with zero hidden layers the ANN is equivalent to logistic regression due to the logistic activation function.

Using weights *W*_1_,*W*_2_,*W*_3_ and biases *B*_1_,*B*_2_,*B*_3_, the ANN forward-propagates from input data *X* to a risk score $\bar{Y}$ by the following three compositions (indicated by parentheses) of the logistic activation function *σ* = *σ*(*z*) having argument *z* (e.g., in the first logistic function, *z* = *B*_1_+*W*_1_*X*),

$$\bar{Y}=\left[\mathrm{risk}\;\mathrm{score}\right]=\sigma \left(B_{3}+W_{3}\sigma \left(B_{2}+W_{2}\sigma \left(B_{1}+W_{1}X\right)\right)\right);\;\sigma (z)\equiv 1/[e^{-z}+1];$$ (1)

We used an in-house MATLAB code to minimize fitting error of (or “train”) our four-layered ANN. Fig 1 shows an example of a four-layer ANN. Our ANN had 12 to 14 inputs, and each hidden layer had the same number of neurons as the input layer. The cross-entropy loss function [37] comparing the model’s predictions, ${\overset{-}{Y}}_{i}$ for the *i*^*th*^ NHIS-respondent’s CRC, with the actual CRC status, *Y*_*i*_ (0 for never-cancer and 1 for CRC), for *N* respondents is,

$$\left[\mathrm{loss}\right]=\frac{1}{N}\ln\prod_{i=1}^{N}{\bar{Y}}_{i}{}^{Y_{i}}{\left(1-{\bar{Y}}_{i}\right)}^{1-Y_{i}}=\frac{1}{N}\sum_{i=1}^{N}\left(Y_{i}\ln{\bar{Y}}_{i}+(1-Y_{i})\ln(1-{\bar{Y}}_{i})\right)$$ (2)

Backpropagation minimizes the fitting error numerically. It involves the chain rule derivative of Eq (1) with respect to weights *W*_1_,*W*_2_,*W*_3_ and biases *B*_1_,*B*_2_,*B*_3_ in iterative gradient decent with the Adam learning rate. Because it numerically minimizes the fitting error Eq (2), backpropagation plays the role of setting slope/intercept-derivatives of the sum-of-squares error equal to zero and solving for slope and intercept in one-variable linear regression.

During training we used ten-fold cross-testing to test our model, while an additional test set (NHIS year 2017 dataset) was held out for developing the stratification scheme after the ANN was trained. Because there is zero intersection [37] between the training and testing datasets, our model has TRIPOD levels [36] of 2a and 2b. The training was done with the standard backpropagation algorithm with the “Adam” learning rate. Rather than selecting a decision boundary to determine what output is considered a positive or negative cancer prediction, we just use the raw output of the ANN of Fig 1 and refer to it, loosely, as a person’s risk of having colorectal cancer.

### Model evaluation

The output of the ANN is a number from 0 to 1 which we treat as a risk-score. Using this output, one can decide on a score-threshold above which the result is considered a positive for CRC and otherwise is a negative result. To evaluate the performance of our ANN, we parametrically plotted the sensitivity and specificity as a function of the risk-score threshold to produce an ROC plot [39,40]. We created an analogous plot with the positive predictive value (PPV) and negative predictive value (NPV).

Stratified cross-testing [41] with ten random folds (having zero intersection [37] with the training dataset) was used to attain a TRIPOD level [36] of 2a. Two sources give statistical variance [37] in the concordance (C) statistic: the first is the number of cancer-cases within the dataset (the population variance) and the second is the number of folds of stratified cross-testing. Hanley and McNeil model the population variance as due to the risk-score being distributed normally (or as a Gaussian). The variance *σ*^2^ is across *n*_*xv*_ stratified cross-validations. The *i*^*th*^ population-variance *σ*_*i*_^2^ and C-statistic *A*_*i*_ is assumed to have a Gaussian distribution and is thus estimated (with maximum likelihood) within a *α*×100% confidence interval for a population of *D* diseased subjects and *H* healthy subjects by [40],

$$\sigma_{i}{}^{2}=\frac{{\left(2\;{\mathrm{erf}}^{-1}\alpha \right)}^{2}}{HD}\left(A_{i}\left(1-A_{i}\right)+\left(D-1\right)\left(\frac{A_{i}}{2-A_{i}}-A_{i}{}^{2}\right)+\left(H-1\right)\left(\frac{2A_{i}{}^{2}}{1+A_{i}}-A_{i}{}^{2}\right)\right)$$ (3)

The total variance *σ*, assuming Gaussian distribution of population-variance [37] and a number *n*_*xv*_ = 10 of folds of cross-testing, is $\sigma^{2}=\sum_{i=1}^{n_{xv}}{\left(\sigma_{i}/n_{xv}\right)}^{2}=\sigma_{0}{}^{2}/n_{xv}$, the average of the variances. Due to the tradeoff of having high variance [37] in the limits *n*_*xv*_→*N* = *D*+*H* (“leave-one-out” stratified cross-testing, where the testing set is just a single data point and thus *D*→{0,1} = 1−*H*) and high bias [37] in the limit *n*_*xv*_ = 2 (“split-sample” stratified cross-testing), the appropriate number of stratified cross-validations must be determined empirically. We thus decided upon ten-fold stratified cross-testing (*n*_*xv*_ = 10).

### Stratifying by risk-score

To demonstrate the potential application of our ANN model in the clinic, we stratified 2017 NHIS respondents [19] into risk-categories. Based on the calculated CRC risk-score from our model, we renormalized and selected a low/medium risk boundary and a medium/high risk boundary to divide the calculated CRC risk into 3 categories. The two boundaries were chosen such that no more than 1% of 1997–2016 NHIS respondents with cancer are categorized as low risk, and no more than 1% of 1997–2016 NHIS respondents without cancer are classified as high risk.

## Results

### Model performance

In Fig 2, the receiver operating characteristic curves (ROC) [39,40] are plotted for the ANN trained with data from Table 2. Cross-testing with ten folds is used to emphasize the generalizability of the training. The sensitivity, specificity, and concordance [18,40] of only the testing across ten random folds (TRIPOD 2a) is reported in Fig 2. The crossed error bars in each ROC are the standard deviation across the ten folds of cross-testing. Inclusion of family history data requires removing a large number of respondents for whom this information is missing, yet gives a performance comparable to the case where all NHIS years are included. It can be seen that deviation from the mean performance of the default model is within 2 standard deviations in the error bars of Fig 2, showing insensitivity to addition or removal of the factors most strongly correlating with being recently and professionally diagnosed with CRC.

At the risk-cutoff value where their sum is maximized, the sensitivity is of 0.57 ± 0.03 and the specificity is of 0.89 ± 0.02 (mean value ± the standard deviation across the ten folds of cross-testing). Uncertainty is larger in the sensitivity compared to the specificity because of the low prevalence of CRC within the dataset. The uncertainty in the concordance has a contribution due to the population [39,40] and a contribution due to the standard deviation across folds of stratified cross-testing [41]. The latter contribution is normally distributed [37] as a result of random shuffling of the data before partitioning into folds of testing.

### Cross-testing between the screened and unscreened

**Fig 3:**
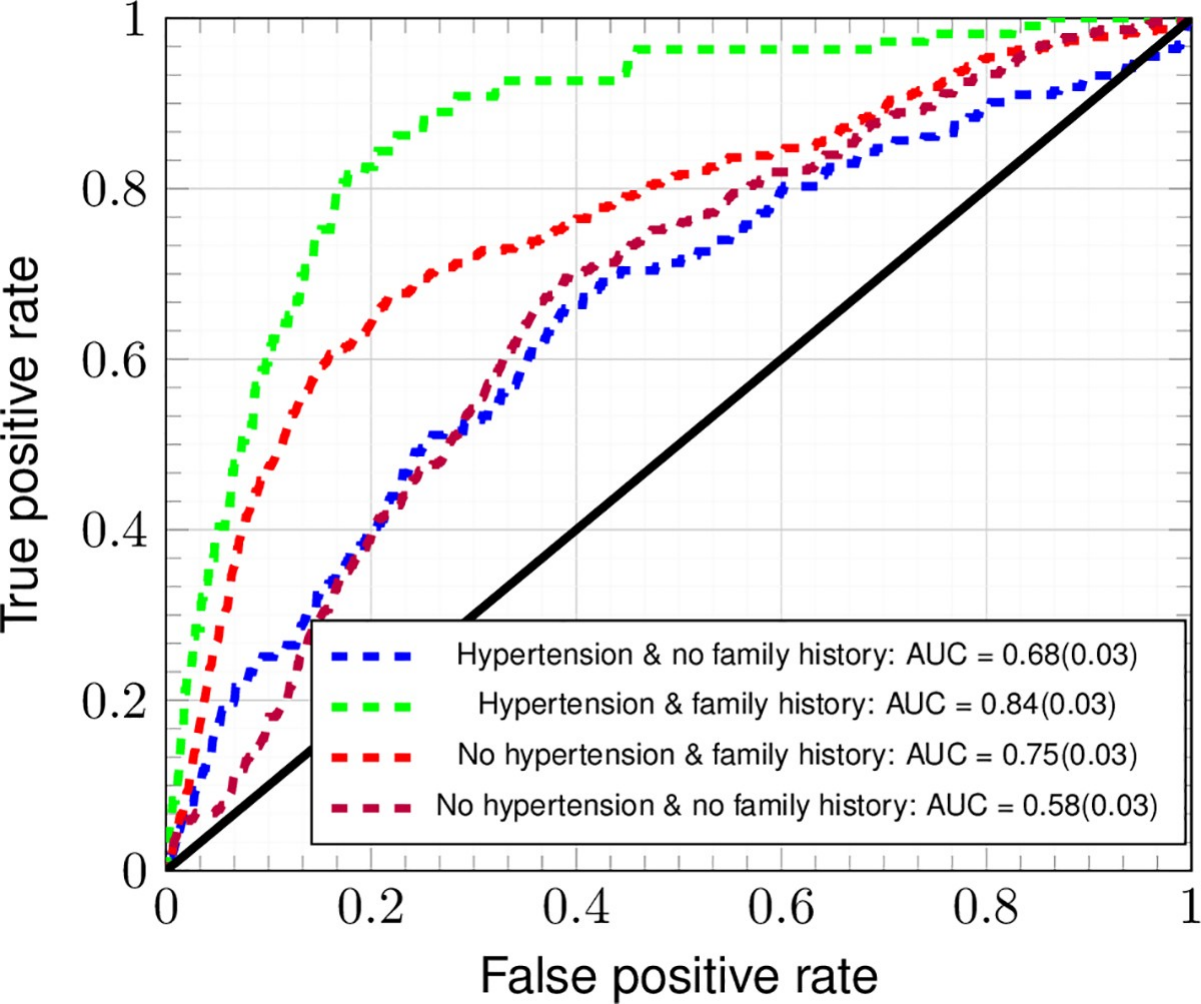
Cross-testing ROC curves of ANN for data non-randomly split between screened and non-screened NHIS respondents (TRIPOD 2b). Using the reduced dataset the ANN was cross-tested between the group of survey respondents (NHIS years 2000, 2005, 2010, and 2015 only) screened for CRC by colonoscopy/sigmoidoscopy and the remaining year group.

In Fig 3 we repeat the analysis of Fig 2, but only use respondents for whom family history data was available. This eliminates any advantage the models without family history gain from having a larger sample size. In addition, we show the performance resulting from cross-testing between the group of those who were ever screened by colonoscopy/sigmoidoscopy and the group formed by the remaining population. The green ROC (hypertension and family history) with an AUC of 0.84 outperforms the blue ROC (hypertension but no family history) with an AUC of 0.68. Similarly, the red ROC (no hypertension with family history) has an AUC of 0.75, better than the 0.58 AUC for the purple ROC (no hypertension no family history). Due to the strong correlation of CRC family history with screening history (see Table 2), inclusion of family history data sharply improves performance by greater than just a standard deviation in testing upon a population not screened by colorectal exam after training on the examined population, as reported in Fig 3. Without family history data, performance worsens by about one standard deviation of true positive rate.

### Performance in age groups

**Fig 4:**
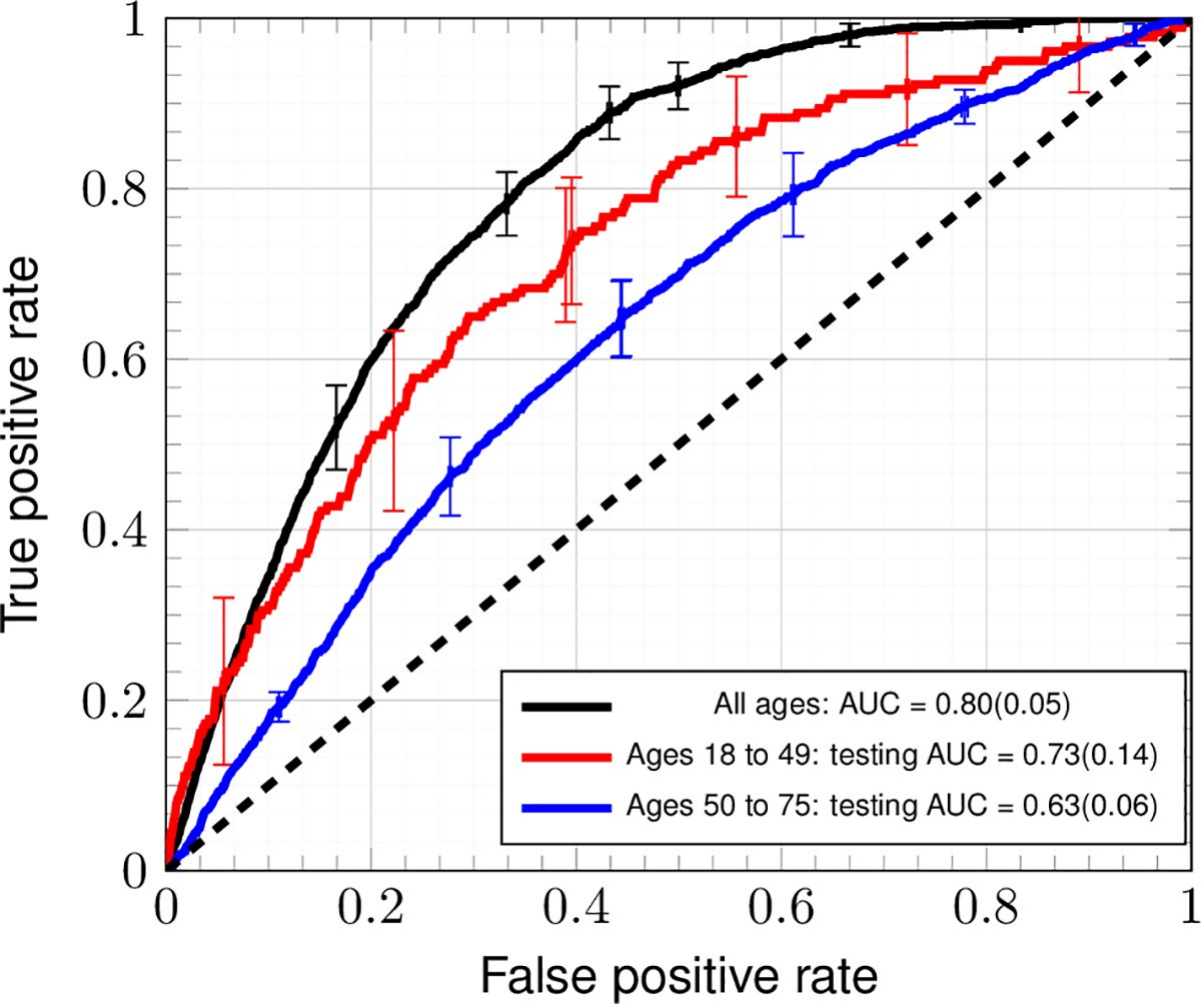
Cross-testing ROC curves of ANN for age groups formed by USPSTF screening guidelines. The ANN is trained by and tested upon 3 datasets: ages 18–49, ages 50–75, and all ages for the full dataset. Error bars denote the standard deviation of the TPR and FPR across ten-fold stratified cross-testing (TRIPOD level 2a).

In Fig 4, ROC plots are given for an ANN trained and tested upon only ages 18–49, ages 50–75, and all ages for tenfold random cross-testing dataset (TRIPOD 2a). These age groups were formed because in ages 50–75 and ages 18–49, USPSTF screening guidelines [22] have respective sensitivity and specificity each of 100% (TRIPOD level 4) in flagging and not flagging a person for screening. There is good [18] mean discrimination in ages 18–49, in which there has been a recent rise in colorectal cancer incidence [11]. In contrast, in ages 50–75 the mean discrimination is only acceptable [18]. The mean is across ten folds of stratified cross-testing [41]. Due to the accompanying standard deviation across folds of cross-testing and the population-variance [40], the performance in ages 18–49 extends down into being merely acceptable, and the performance in ages 50–75 extends down into being indiscriminate [18]. Clearly, performance in cross-testing within ages 18–49 is better than the performance in cross-testing within ages 50–75.

### Predictive value of the ANN

**Fig 5:**
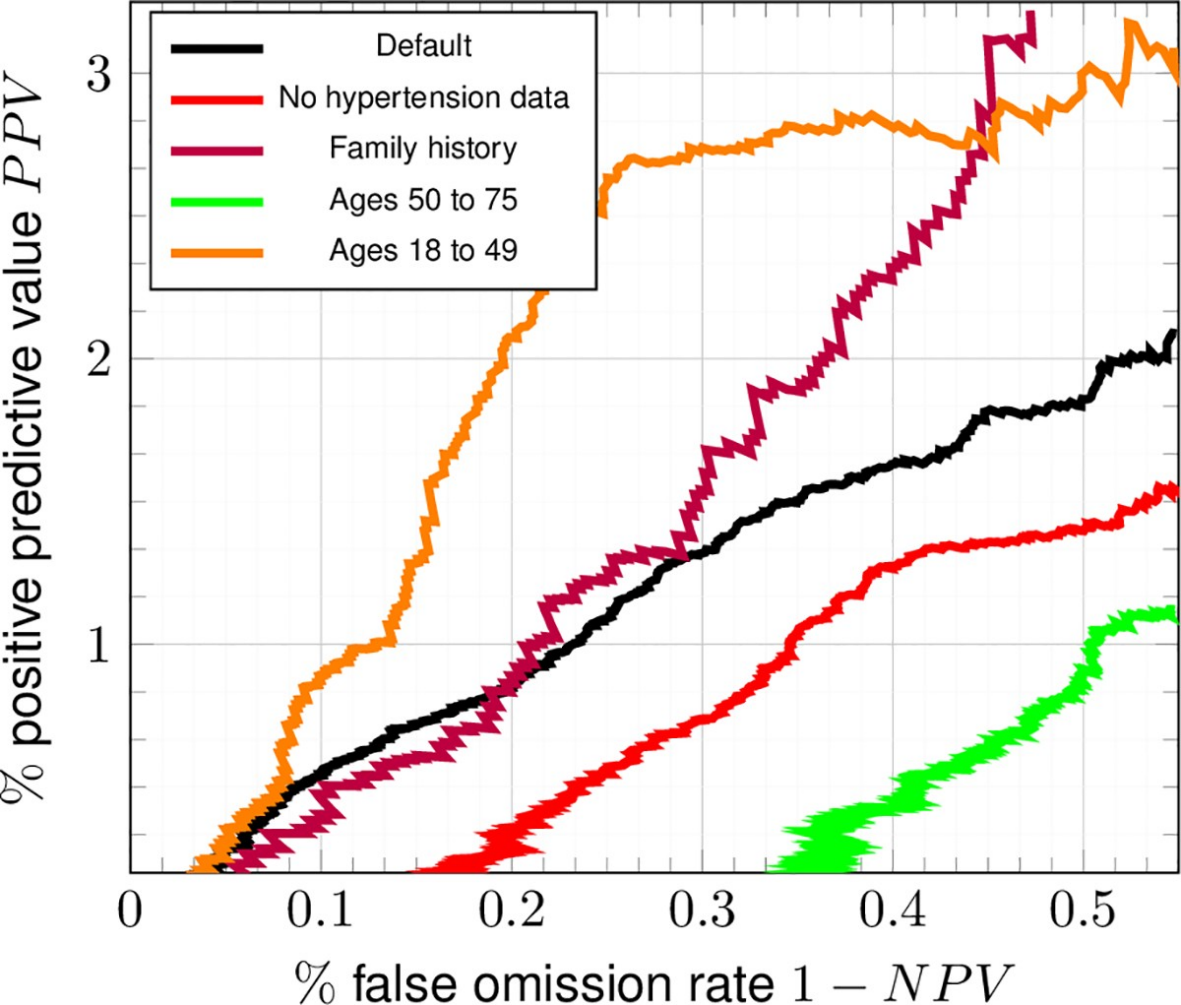
Diagnostic performance of the ANN for the random testing dataset (TRIPOD level 2a). Positive predictive value PPV and false omission rate were parametrically plotted in analogy to Fig 2.

In Fig 5, the positive predictive value and false omission rate is reported for a wide variety of risk-cutoffs. The positive predictive value of the trained ANN is much lower than its negative predictive value at almost all values of the risk-cutoff, meaning that a negative call by the ANN is far more meaningful than a positive call [37]. Due to the NHIS dataset being a cross-sectional study with no follow-up, it remains possible that the false positives that drive the PPV down to such a low value are those who are of high risk and have CRC that has not yet been detected (whom it is highly desirable to screen). Barring this possibility, the ANN is better suited for making screening recommendations than functioning as a diagnostic tool, and thus is next demonstrated to calculate risk.

### 3-category risk stratification

**Fig 6:**
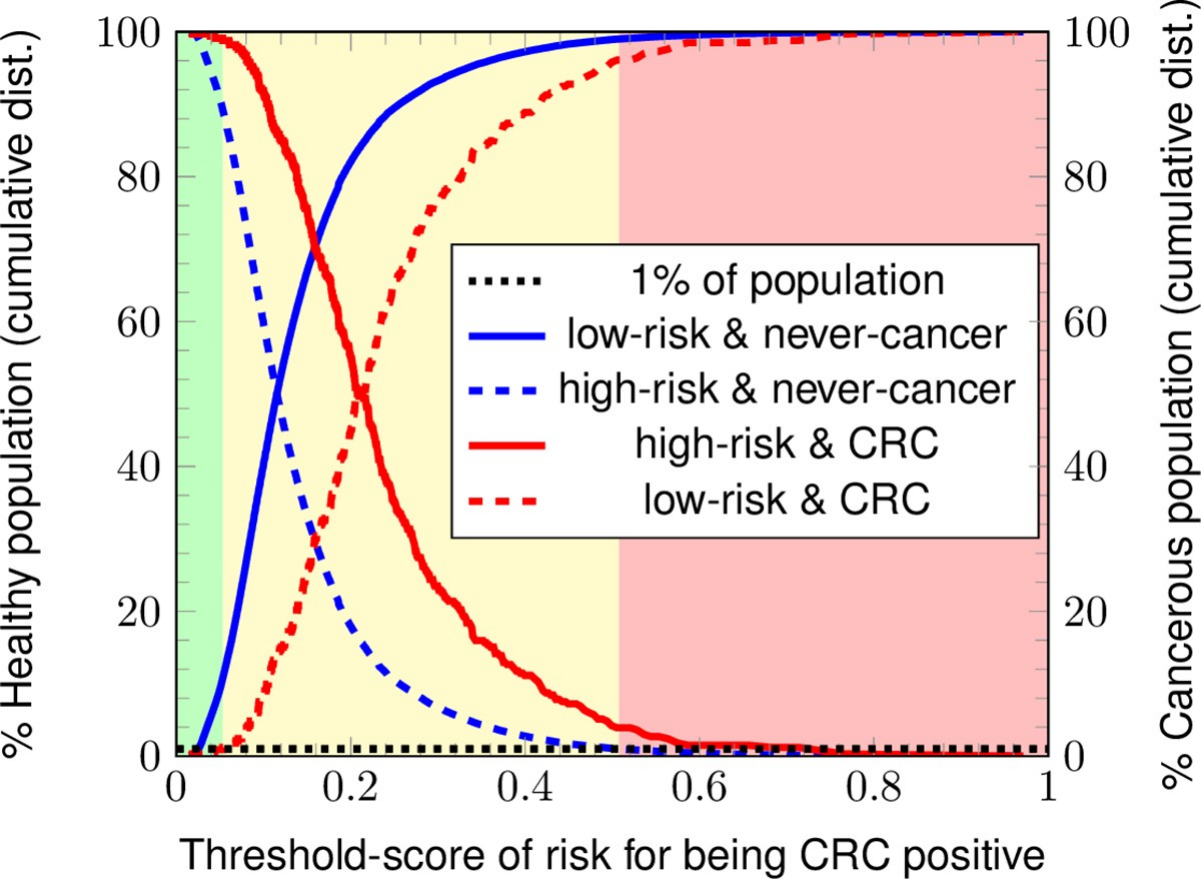
Risk stratification into three categories. The 2017 NHIS respondents are stratified by the ANN into three categories for CRC risk: green (low risk), yellow (medium risk), and red (high risk).

To test its potential application in stratifying colorectal cancer risk, we ran the developed ANN with the 2017 NHIS dataset [19]. As shown in Fig 6, three categories of risk (green: low risk; yellow: medium risk; red: high risk) are stratified. The solid-lined cumulative distributions are the respective cancer/non-cancer 2017 populations correctly classified as high/low risk. The dashed-line cumulative distributions are the respective cancer/non-cancer 2017 populations misclassified as low/high risk. The low/medium and medium/high risk boundaries are defined by requiring a 1% misclassification rate for the 1997–2016 NHIS respondents. The CRC and never-cancer 2017 NHIS respondents are misclassified at respective rates of 5% and 6%.

A summary of the results of stratifying the populations into three risk categories is given in Table 3. Our ANN classifies 8% of the cancerous population as high score and 87% of the same as medium risk with a misclassification rate of 5%. For the non-cancerous population, our ANN classifies 12% of the non-cancer population as low score and 82% of the same as medium risk with a misclassification rate of 6%. In comparison, the USPSTF guidelines misclassify 35% of the cancerous population as low risk and 53% of the non-cancerous population as high risk at TRIPOD level 4.

**Table 3:** Comparison of ANN risk-scoring with USPSTF screening guidelines on 2017 NHIS dataset for 3-category risk-score stratification.

	# Respondents	# Low Score	% Low Score	# Medium Score	% Medium Score	# High Score	% High Score
**Our ANN**							
**CRC (2017)**	60	3	5%	52	87%	5	8%
**Never-cancer (2017)**	25,457	2,932	12%	20,998	82%	1,527	6%
**USPSTF Guidelines**							
**CRC (2017)**	60	21	35%	n/a	n/a	39	65%
**Never-cancer (2017)**	25,457	11,845	47%	n/a	n/a	13,612	53%

## Discussion

Due to the low value of its positive calls, the ANN will not replace current screening methods for CRC. This can be seen by the comparison in Table 4 of the PPVs among conventional screening methods with that of the trained ANN found in Fig 5. The gold standards of colonoscopy and sigmoidoscopy are the only tests with PPV close to 1, but these tests are invasive and sometimes injurious [22]. Their lower-PPV counterparts, FIT, FOBT, and SEPT9, have varied test accuracy depending on the CRC stage [42] (a feature shared by colonoscopy and sigmoidoscopy, albeit to a lesser extent). Different levels of risk [43] as in Fig 6 could be assigned different screening methods, depending on the judgment of the clinician and the availability of resources (e.g., in some countries, the FIT is the only screening test available). An example of such a scheme for concreteness: a clinician might preferentially give colonoscopies (most expensive) to those of high risk, SEPT9 and/or FOBT tests (moderate expense) to those of medium risk, and FIT (least expensive) to those of low risk (see Table 4) [44,45]. Given the low cost, ease of mass implementation, and low invasiveness of the trained ANN, it emerges as highly attractive for stratifying risk of CRC, despite its inability to perform screening.

**Table 4:** Comparison of ANN to conventional screening methods.

Screening method	Sensitivity, Specificity and/or PPV	Advantages	Disadvantages
Artificial neural network (ANN) trained with NHIS data years 1997–2016 tested on ten random splits	● Sensitivity ~ of 0.57 ± 0.03 ● Specificity ~ of 0.89 ± 0.02 ● PPV ~ of 0.0075 ± 0.0003	● Better performance w/more training data ● Privacy ● Inexpensive ● Stage-independent ● Can stratify risk	● Low PPV ● Assumes integrity of data ● Only correlation ● Cannot be used for screening
Guaiac or immunoassay fecal occult blood test (gFOBT or iFOBT)	● Sensitivity ~ 0.9 ● Specificity ~ 0.9 ● PPV ~ 0.02	● No pre-test colon-cleansing ● Privacy ● Non-invasive	● Low PPV ● Pre-test diet ● False-positives ● Depends on CRC stage ● Moderately expensive
● Fecal immunochemical test (FIT) ● Fecal immunochemical DNA test (FIT-DNA)	(1) For FIT: ● Sensitivity ~ 0.1 ● Specificity ~ 0.9 ● PPV ~ 0.4 (2) For FIT-DNA: ● Sensitivity ~ 0.2 ● Specificity ~ 0.9 ● PPV ~ 0.5	● No pre-test colon-cleansing ● Privacy ● Inexpensive ($14) ● Non-invasive	● Adenoma insensitivity ● False-positives ● Low PPV ● Depends on CRC stage
Methylated SEPT9 gene test	● Sensitivity ~ 0.6 at Stage I. ● Sensitivity ~ 0.9 at Stage IV.	● No pre-test colon-cleansing ● Privacy ● Noninvasive	● Moderately expensive ● Depends on CRC stage
Flexible sigmoidoscopy	● Sensitivity ~ 0.6 ● Specificity ~ 0.7 ● PPV ~ 0.8	● Able to perform biopsy/polypectomy ● Less colon-cleansing ● No sedation	● Only rectum, lower-colon ● Dieting, bowel cleansing ● Invasive ● Expensive
Virtual colonoscopy	● Sensitivity ~ 0.6 ● Specificity ~ 0.7 ● PPV ~ 0.8	● Noninvasive ● Sedation unneeded ● Better at identifying advanced adenomas.	● Colon-cleansing ● Ionizing radiation ● Expensive (~$8000 in costs and charges)

There are several aspects of our chosen methods that minimize the effects of potential sources of bias in the calculated risk. While the ANN is a powerful statistical tool [46], the ANN is only as good as the data used to train it, so a discussion of these biases is called for.

First, the outcome variable is defined as those NHIS respondents [19] recently and professionally diagnosed with CRC, and not necessarily those cases of CRC diagnosed by the gold predictive standard of colonoscopy (see Table 4). Unfortunately, NHIS years 2000, 2005, 2010, and 2015 only contain data on whether the respondents have ever been screened by sigmoidoscopy, colonoscopy, or proctoscopy. In lieu of such screening data for every year, Fig 3 reports testing-performance of a model trained on the screened population and tested on the remaining population. Performance without family history is within a standard deviation of true positive rate poorer, and is significantly greater with training by family history (which correlates extremely strongly with having been screened). The decrease in performance without family history data is attributable to the biases and confounders we discuss.

Another concern is that the predictor variables shown in Table 2 are marked as “ever” having occurred, while the age at which the NHIS respondent was professionally diagnosed with CRC is recorded. This is the purpose of the 4 years cutoff beyond which an instance of CRC is regarded as too long ago from the date of taking the NHIS, and is discarded, thus decreasing (if not eliminating) the probability that the predictors came before the CRC diagnosis. Previous work scoring risk of lung and skin cancer [43,47] have found their ANN insensitive to this cutoff.

The correlation of a given factor with CRC incidence (see Table 2) is a necessary but insufficient condition for that factor to be definitely causal of CRC. This is why the effect of training with and without hypertension data in the training of the ANN is indicated in Fig 2. Patients taking bevacizumab [3] for CRC often develop high blood pressure [4,5], and if this was largely responsible for the high correlation found in Table 2 it would be inappropriate to train the ANN with this. As it turns out, bevacizumab was FDA-approved [38] as a second-line treatment of metastatic CRC in 2004 and the NHIS dataset [19] of personal health data extends from years 1997–2017. The correlation of hypertension with CRC in years 1997–2003 is 3.25×10^−2^ and in years 2004–2017 decreases (unexpectedly) to 2.97×10^−2^. It is thus possible that the predictor variables in Table 2 have their confounding with those NHIS respondents recently and professionally diagnosed with CRC controlled to some degree. This allows inclusion of hypertension as a predictor, which is important due to the role of hypertension in CRC recurrence and mortality [48,49].

Another source of bias lies in what age the NHIS respondent was recently and professionally diagnosed with CRC. There is a bias towards screening at that age interval because this age interval was selected by the USPSTF [22], and this is seen on a histogram of CRC incidence vs. age of diagnosis where it is observed that about 60% - 70% of CRC cases occur in ages 50–75. Clearly, the diagnosis came at the time of screening, so the screening time could be far after the time the CRC first nucleated. This may call for an augmentation of the NHIS survey with a question about the extent of the CRC’s advancement when it was first detected by screening.

The last source of bias is due to discarding any NHIS respondent with one or more blank entries as a result of answers of “refused”, “not ascertained”, or “don’t know” (see NHIS documentation [19]). It is speculated that responses of “not ascertained” and “don’t know” belong to data missing completely at random, while the response of “refused” belongs to data missing at random [50]. For instance, an answer of “refused” to the factor of a subject’s smoking habits may mean smoking of illegal substances. If this is true, then our model would carry the bias [37] of excluding (and thus less accurately scoring CRC risk in) the corresponding members of the population. A similar bias results from the model excluding those having CRC more than 4 years from the year they answered the NHIS, which are also discarded. Future work would draw upon imputation techniques allowing for systematic treatment of such missing entries, thus avoiding the loss of an entire survey-respondent. This is especially critical in fields with larger quantities of missing data, such as family history of CRC heavily relied upon by clinicians to make screening recommendations.

We now discuss the ANN architecture. An ANN with two hidden layers is selected due to its being the smallest ANN that can learn low-degree polynomial functions [51]. This allows the ANN to deal with noise. The ANN’s good performance in spite of the bias and confounding discussed above could be attributable to it viewing these as noise. The cross-tested C-statistic of logistic regression [18] (ROC not shown) is 0.60 ± 0.03, suggesting the importance of inter-factor coupling whose incorporation is made possible by a second hidden layer [21]. In the ANN, factors are fed into any one trained neuron of the first hidden layer, linearly combined by the weights, and composed with the sigmoid function. Each neuron of the first hidden layer thus takes on a value that is the pooled effect of all factors of the input-layer. Each neuron of the second hidden layer receives these various pooled values, and thus is the layer where the trained weights incorporate inter-factor couplings before being fed into the single output-neuron (CRC risk score). If the second hidden layer is made of a single neuron, it becomes equivalent to the output neuron and the calculated risk incorporates no inter-factor coupling. This gives a C-statistic of ~0.5, which means the ANN is almost maximally indiscriminant [18]. We interpret this (as well as the insensitivity of the C-statistic to presence/absence of data on hypertension and family history reported in Fig 2 and Fig 5) as indicative of how much more important inter-factor coupling in one’s risk of CRC is compared to factors acting in isolation (which the process of naïve one-by-one variable selection assumes). The insensitivity of the ANN to being trained or not trained with cancer-family-history data (NHIS years 2000, 2005, 2010, and 2015 only) shown in Fig 2 might also be attributable to inter-factor coupling [21].

A model of CRC risk within the NHIS dataset [19] has been developed thusly. The model shows predictive power in a general demographic in Fig 2. The resulting concordance is 0.80 ± 0.05, and thus is competitive with that of Kaminski et al.[15] (which incorporates family history of CRC as well as regular aspirin use [52]) and with that of the highest-performing models (among 11) [15] in a recent review of MEDLINE, Scopus, and Cochrane Library databases from January 1990 through March 2013 [17]. In Fig 3, because performance improves by greater than one standard deviation of true positive rate on including family history data and worsens by one standard deviation of true positive rate, the worsening of performance can be attributable to the sources of bias described above (taking colorectal exams to be the gold standard of diagnosis as in Table 4). The trained ANN retains its predictive power even within ages 18–49: in Fig 4, the standard deviation of true positive rate approximately reaches the performance at all ages. This suggests promise in performance in the demographic not flagged for screening by age. Although the ANN’s positive calls are almost meaningless (see Fig 5), it is usable as a risk stratification tool (see Fig 6 and Table 3) to assist clinicians.

Future work will study generalizability. The present work omits a calibration plot, and just report discrimination due to this being a development (rather than validation) study. Future work will study training/testing with the NHIS data and testing/training upon a separate dataset to determine the effect of disparate risks of CRC between datasets upon model performance. Advanced techniques such as validation-based early stopping and/or dropout are also to be used.

A future aim is to implement this system of risk scoring in a software-application (an “app”) that a smartphone can run. The application will be along the lines of CT Gently [53], an application previously developed. The user of the application will answer NHIS survey questions and immediately receive the scoring of their risk for CRC, in much the same manner as a current website [54] hosting an algorithm underpinning the corresponding published [55] model. Moreover, the application will simulate immediate adjustments in the user’s personal health habits: for instance, the user will be able to drag a sliding-bar representing their smoking habits from high to low and see their score of CRC-risk drop in response to quitting smoking. Alternatively, the ANN could retrieve electronic medical records in a clinical setting and provide immediate risk-scoring during consultation.

## Conclusion

A multi-parameterized artificial neural network was developed to score risk of colorectal cancer based solely on personal health data. The trained ANN has been robustly tested per TRIPOD level 2a and level 2b protocols. The concordance of the ANN is comparable to that of current methods of scoring CRC risk (including those using biomarkers). The ANN outperforms logistic regression, suggesting the importance of inter-factor coupling. The low positive predictive value indicates unsuitability of the ANN to replace conventional screening methods. Nevertheless, in comparison to USPSTF guidelines, the trained ANN can stratify individual’s colorectal cancer risk more accurately for more effective screening and intervention. As the ANN is built from self-reportable personal health data, it can be easily implemented on a mobile platform for more widespread applications.
